# Neuro syphilis: Portrayals by Sir Arthur Conan Doyle

**DOI:** 10.4103/0019-5545.55103

**Published:** 2009

**Authors:** O. Somasundaram

**Affiliations:** Thanigai Illam, 23^rd^ Cross, 30 Besant Nagar, Chennai, India

**Keywords:** Neurosyphilis, history, fiction

## Abstract

The developments in neuro syphilis in the 19^th^ century are integral parts of the history of psychiatry. The delineation of various aspects of neuro syphilis by Sir Arthur Conan Doyle in three of his stories is discussed in brief.

The medical profession is partial to the literature of medical writers. Sir Arthur Conan Doyle,[1] the author of *The Adventures of Sherlock Holmes,* etc., W. Somerset Maugham (1874-1961), the author of *Of Human Bondage, Cakes and Ale,* etc., A. J. Cronin (1896-1981), the author of *Citadel, The Keys of the Kingdom,* etc., and Anton Chekhov (1860-1904), the author of many short stories and *Cherry Orchard* (Chekhov once said: “Medicine is my lawful wife and literature is my mistress”) are some of the famous medical writers. The medical men see their personal and professional life depicted in many of their stories, and it is not surprising that many medical illnesses are portrayed in minute details.

Conan Doyle is no mean exception to the aspect not only of the medical knowledge but also the psychological aspects of the mind that are replete in his stories. The following article narrates three stories related to neurosyphilis. His interest in and intimate knowledge of the disease can be gauged from his doctoral thesis on tabes dorsalis in 1885 in the University of Edinburgh. It is also interesting to note that his father Charles, an artist, had alcohol problems which necessitated his admission into the Montrose Royal Lunatic Asylum, the Edinburgh Royal Infirmary, and finally at Crighton Royal Institute where he died in 1893. Conan Doyle's familiarity with general paralysis of the insane (GPI) and tabes dorsalis should have increased enormously during his visits to the various asylums.

Before entering into the fiction of neurosyphilis, it is worthwhile recalling the history of neurosyphilis. Once called “the disease of the century,” neurosyphilis has been virtually forgotten today and is routinely ignored by historians of psychiatry, according to Edward Shorter.[2] He also gives a concise history of this disease.[3]

Among its celebrated victims of the 19^th^ century are Schumann (German composer, aesthete, and music critic), Maupassant (French writer), Donizetti(Italian composer), and Nietzsche (German philosopher and philologist). Clinical aspects of Maupassant are discussed by the author in an earlier article.

Hare[4] elaborately discusses the origin and spread of dementia paralytica. In spite of the widespread prevalence of syphilis in the Middle Ages, the central nervous system was not known to be involved till the early decades of the 19^th^ century. This is attributed to a mutant neurotropic “syphilitic virus” strain, which spread from northern parts of France to Europe and other parts of the world. The descriptions of GPI are attributed to English physicians like Thomas Willis. John Haslam of the Bethlehem Hospital before this period is not given credence. Instead, Bayle (1882) Calmeil (1826) were given. Between January 1815 and July 1823 Bayle collected 189 cases of chronic meningitis (i.e., dementia paralytica) among 847 male admissions and 25 among 606 female admissions to Charenton.

Let us now go into Conan Doyle's fictional world.

## TALE NO. 1: A MEDICAL DOCUMENT - THE CLASSICAL GRANDIOSE TYPE OF G.P.I.

Charley Manson, Chief of Wormley Asylum gives the study of his patient:

“Well, take a common complaint which kills many thousands every year like G.P. for instance.”

“What is G.P.?”

“General Practitioner”, suggests the surgeon with a grin.

“The British Public will have to know what G.P. is” says the alienist gravely.

“It's increasing by leaps and bounds, and it has the distinction of being absolutely incurable. General Paralysis is its full title and I tell you it promises to be a perfect scourge. Here's a fairly typical case now which I saw last Monday week. A young farmer, a splendid fellow surprised his fellows by taking a very rosy view of things at a time when the whole country side was grumbling. He was going to give up wheat, give up arable land, too, if it didn't pay; grow a thousand acres of rhododendrons and get a monopoly of the supply for Covent Garden … There was no end to his schemes all sane enough but just a bit inflated … Something about the man's way of talking struck and I watched him narrowly. His lip had a trick of quivering, his words slurred themselves together and so did his handwriting when he had occasion to draw up a small agreement. A clear inspection showed me that one of his pupils was ever so little larger than the other … but I know the fellow was as much condemned to death as though he were lying in the cell at Newgate. It was a characteristic case of incipient G.P.”

**Comments**

At the time of Conan Doyle writing this story in 1894, there was almost an epidemic of GPI in the English asylums.
Steward[5] gives the figures as shown in Table 1.

**Table 1:** Admissions of GPI pattents in English asylums

Years	Male	Female	Total
1878-82	12.8	3.3	8.0
1883-87	14.3	3.1	8.0
1888-92	14.7	3.4	8.8
By 1849, Conolly finds that among 690 deaths at Hanwell over last 10 years, the proportion of these due to general paralysis was 37% in males and 11% in females. These figures for a London asylum were probably well above the average for the country, as in 1876.
The position was not different in Scotland. The incidence of GPI in all asylums of Scotland was officially given as 18% in males and 3% in females (Commissioners in Lunacy for Scotland).
The delusions in GPI were characteristically grandiose in the early 19^th^ Century. Bayle's patients govern the universe, have 40 million tons of gold, own marble palaces or build a new paradise. Calmeil (1826) considered that ideas of grandeur were present in “a very great number” of insane paralytics. Conolly (1849) says,” The disease is so generally associated with ideas of wealth and grandeur that when these prevail strongly in any patient we may expect the paralysis to supervene. It is scarcely ever combined with melancholia.” In 1871, Blandford, a lecturer in psychological medicine at St.George's Hospital, still taught that “almost all, certainly 19 out of 20, paralytics are full of ideas of their greatness, importance and riches.”
6. F. W. Mott was the Director of Pathological Laboratory of London County Asylums during this period, and has given an elaborate account of the pathology and diagnosis of neurosyphilis.[6]

## TALE NO. 2: THE SURGEON TALKS - A TALE OF TABES DORSALIS

Dr. Walker of St. Christopher's was one of the best men in Europe on nervous disease. He is the author of a little book on sclerosis of the posterior columns. It's as interesting as a novel and epoch-making in its way. He worked like a horse, spent many hours a day in the clinical wards. He had a huge consulting practice. Walker was a single man which means that he was not restricted to a single woman.

Walker was lecturing on locomotor ataxia to a wardful of youngsters. He was explaining that one of the early signs of complaint was that one of the early signs of complaint was that the patient could not put his heels together with his eyes shut without staggering. As he spoke, he suited the action to word.

This is the first time Walker realized that he was positive for Romberg's sign. His clinical assistant confirmed that his lower limb deep jerks were totally absent. Walker knew that what he suffered from was not intercostal neuralgia but the lightning pains of tabes. Clinical diagnosis of tabes was established and Walker accepted the inevitable fate… it took five years to kill him and he stood it well. If he had ever been a little irregular he atoned for it in that martyrdom. He kept an admirable record of his own symptoms and worked out the eye changes more fully that has ever been done. When his ptosis got very bad he would hold his eyelid up with one hand while he wrote. Then, when he could not co- ordinate his muscles to write, he dictated to his nurse. So died, in the odour of science, James Walker at 45.

**Comments**

7. In 1814, Christian Friedrich Harless, then Professor of Medicine in Erlangen, referred to the spinal version of neurosyphilis. Three decades later, the great Berlin neuropathologist Moritz Romberg observed that this disease had recently increased “following the great military campaigns of our era.” He baptized it tabes dorsalis.[2]

## TALE NO. 3: THE THIRD GENERATION – CONGENITAL NEURO SYPHILIS

This story is admirably summarized by Turner:[7]

This describes the visit on a rainy evening, of a young aristocrat to Dr. Horace Selby “a specialist who has a European reputation.” The young man shows the doctor a lesion on his shin, and when asked to “account for it” insists that he had done “nothing in my life which to reproach myself.” The lesion is described as “serpiginious;” he is then asked to show his teeth (“at which the doctor again made the gentle clicking sound of sympathy and disapprobation”) and his eyes are examined. The doctor's “pleasure” after this is described as the kind of enthusiasm “the botanist feels when he packs the rare plant into his tin knapsack.”

The case is declared as “typical;” the patient is told that he has “interstitial keratitis” and that there are “indications of a strumous diathesis.” It is considered that he has “a constitutional and hereditary taint” – the father and grandfather had had a similar condition, the latter being “a notorious buck” of the 1830s who apparently “died horribly” and the doctor now considered that his deeds were “living, and rotting the blood in the veins of an innocent man.” The patient recalls that his father always wore gloves in the house, worried about “his throat, and then his legs,” and that he was constantly fussing about his son's health.

When advised by the doctor that there were “many thousands who bear the same cross as you do,” he bursts out with anger at the injustice of it all, and confesses he is soon to be married. They discuss ways in which he might postpone this event (such as an urgent visit to Australia), and various powders and ointment are prescribed. The next morning, the doctor wakes to read in the newspaper of “a deplorable accident.” A young man had “slipped and fallen under a heavy carriage and died before reaching hospital. This young man, of course had general paralysis of the insane (GPI) with the hereditary stigmata of Hutchinson's teeth and an Argyll–Robertson pupil.

**Comments**

8. Steward[8] discusses congenital paralysis and the above story illustrates this variety of paresis.

9. The relationship between syphilis and mental retardation has been described in an earlier paper.[9,10]

## CONCLUSION

The disease, general paralysis of the insane which reigned supreme in the asylums of the 19^th^ century has almost disappeared from the scene. This can be compared with the eradication of small pox in most parts of the world. The pride of place is now given to HIV/AIDS in the present century. Can we look forward to its disappearance in the course of the century?
